# A Method of Strengthening Vulcanite Plates

**Published:** 1897-01

**Authors:** C. R. Morley

**Affiliations:** L.D.S. (Eng.)


### Abstracts and Translations.

### A METHOD OF STRENGTHENING VULCANITE PLATES.

BY C. R. MORLEY, L.D.S. (ENG.)

THE following are the details of this demonstration:

Having obtained your plaster model in the usual way, take a zinc die with a lead reverse. Upon zinc strike a tin plate, No. 8 or No. 10, the size intended for the finished work, try this in the mouth, and, having got a correct adaptation, take the bite, make articulation, and proceed to set up pin teeth in the ordinary way. Afterwards try temporary plate and teeth in the mouth and ascertain that the bite, position of the teeth, etc., are correct; then place plate upon the model and take plaster impressions of the fronts of the teeth. Take the temporary plate to pieces, and the relative positions of the teeth and model to each other will be given. The next step is to strike up a copper plate, No. 3, and upon this copper plate strike a perforated dental alloy plate, No. 6, and upon this a No. 8 or No. 10 tin plate, guided by the fronts and teeth; these plates must be made of such a size that they will just clear the pins of the teeth and lift out of the flask. Round the perforated dental alloy plate, under or a little within the pins of the teeth, a strip of strong plain dental alloy plate should be soldered at "*right angles*." The perforated dental alloy plate should extend from first molar to first molar, taking care to end the bar between the pins of either the first or second molars, and not opposite the division between teeth. At this stage the palatal portion of the plaster model is covered with a copper plate, and upon this a strengthened perforated dental alloy plate, and upon this again a tin plate; upon these plates wax up the teeth to the same extent, and in the same way as if attaching teeth by means of vulcanite to a gold plate. Then proceed to flask, and in ordinary cases use a two-part flask. "The packing is very important," and care must be exercised at every step. Begin by packing under and between the teeth in the usual way, then take a piece of extra thin rubber, cut it to the size of perforated dental alloy plate, place under the perforated plate, that is, upon the palatal side, and afterwards replace upon model in exact position, place tin plate upon this, heat up flask, and gradually close it. Now open the flask, remove the plate, and if the operation has been successfully conducted the perforated plate will be in the same position upon the model as before, only the copper plate is replaced with rubber; finish the packing and vulcanize in the usual way. In partial uppers the plan may be modified by substituting for the rectangular bar a piece of plain dental alloy plate, No. 8, about one-eighth or one-quarter inch wide, struck upon the labial edge of perforated dental alloy plate and soldered to it. In getting up and polishing plate care must be taken to avoid exposing perforated plate. Although this mode of working has been given as "a method of strengthening vulcanite plates," it could more correctly be described as "a method of making plates, using vulcanite as a means of gaining the fit to the mouth, and of attachment for the teeth." Stress must be laid upon the leading feature, that practically the whole strength is the strengthened perforated dental alloy plate so arranged as to give the greatest degree of strength with the least substance.

